# Supplementary material for: A bacterial defense system targeting modified cytosine of phage genomic DNA
Source: Nat Commun. 2026 Jan 22;17:1920. doi: 10.1038/s41467-026-68792-8 (PMC12923530; doi:10.1038/s41467-026-68792-8)
Supplement: Supplementary file 1 — Supplementary information [file 41467_2026_68792_MOESM1_ESM.pdf]

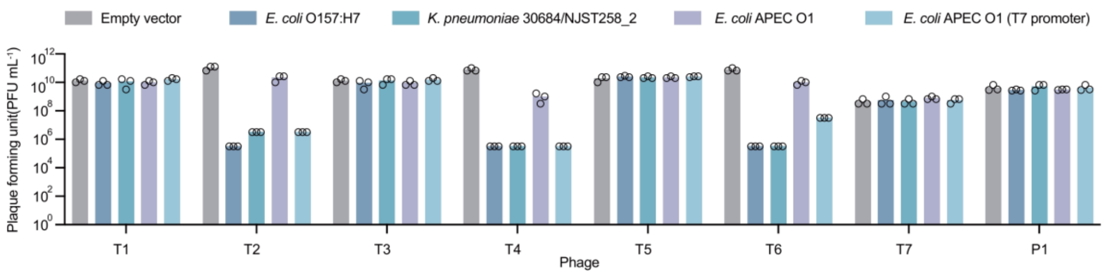

Supplementary Figure 1. Phage resistance of CMoRE system. Anti-phage activities of the *E. coli* BL21(DE3) strain containing empty vector or CMoRE system against various phages are measured by plaque formation assays. Data represent plaque-forming units (PFUs) per milliliter of each phage infection. Bar graphs show the average of three replicates with individual data points overlaid. Source data are provided with this paper.

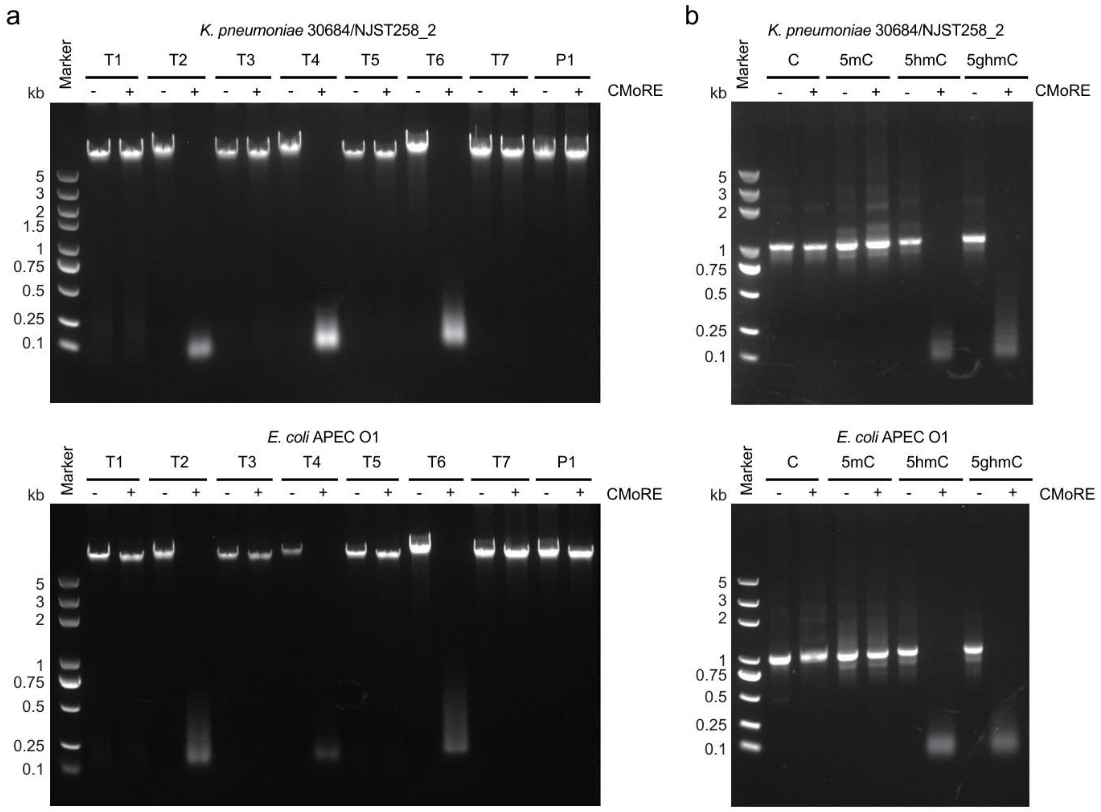

Supplementary Figure 2. The endonuclease activity of the CMoRE systems from *E. coli* APEC O1 and *K. pneumoniae* 30684/NJST258_2. (a) CMoRE degrades the genomic DNA of T-even phages. (b) Endonuclease activity of CMoRE against various cytosine modifications. Source data are provided with this paper.

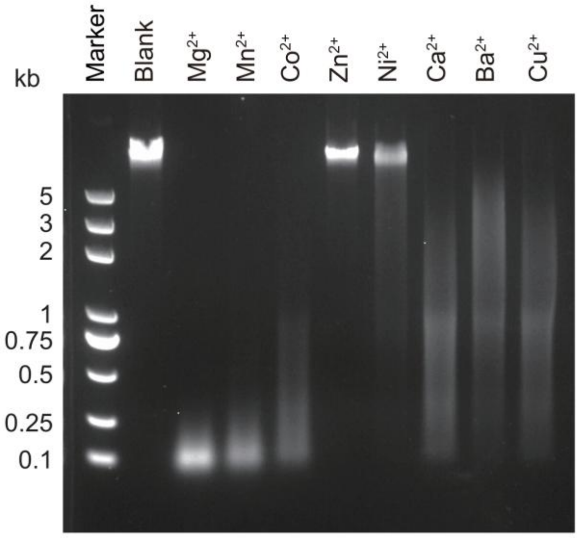

Supplementary Figure 3. The metal dependent endonuclease activity of CMoRE against T4 genomic DNA. Source data are provided with this paper.

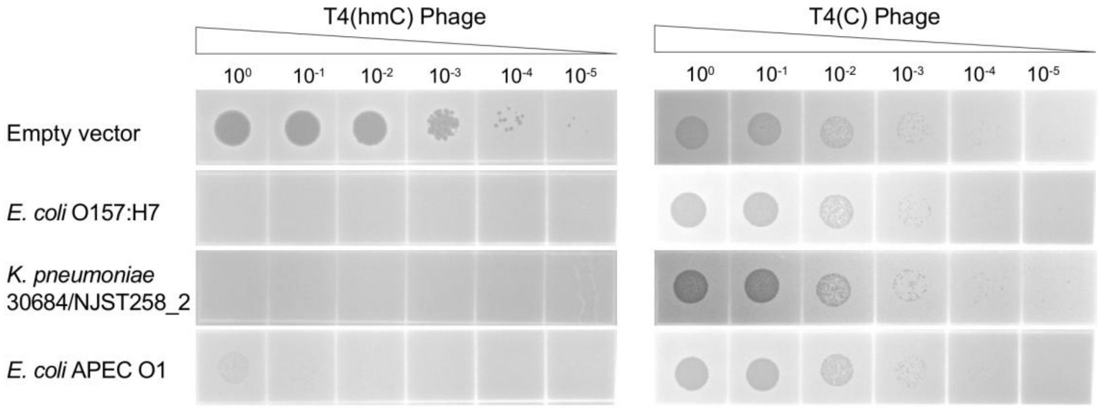

Supplementary Figure 4. Plaque formation assays on CMoRE defense systems from three bacterial species against two genetically engineered T4 phages. The empty vector is used as the negative control. Source data are provided with this paper.

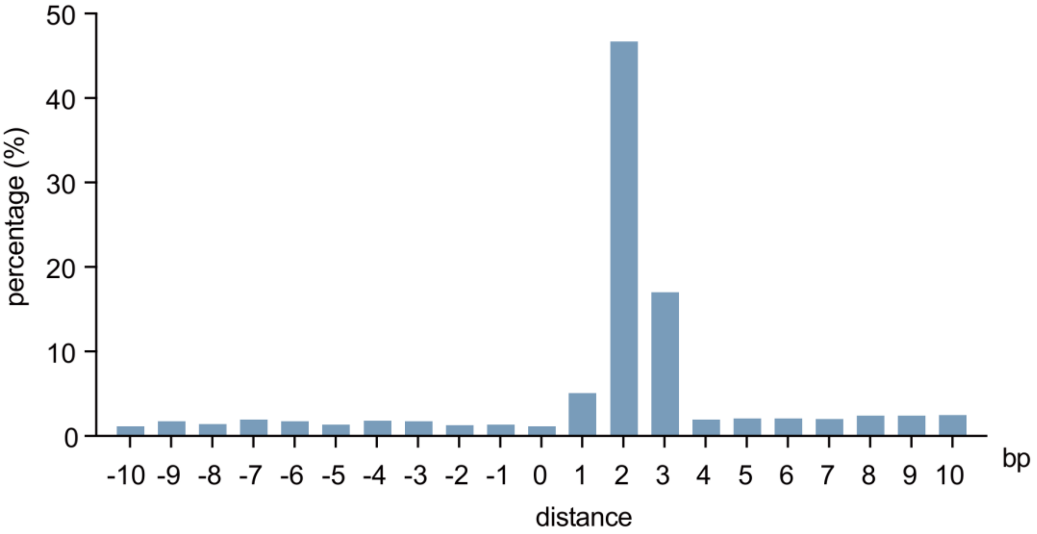

Supplementary Figure 5. Calculated distance between the mapped digested DNA products.

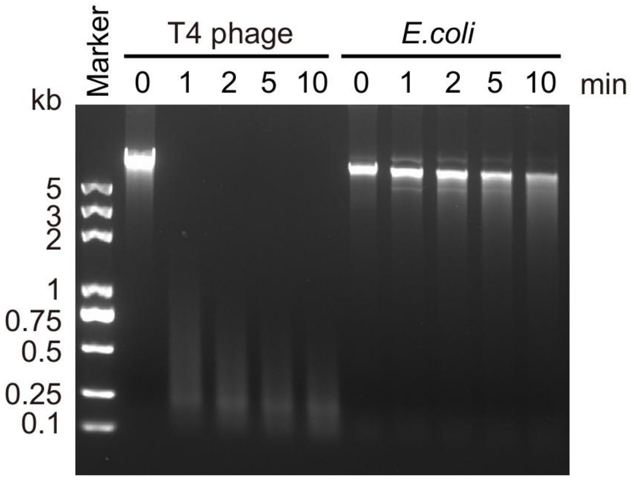

Supplementary Figure 6. Endonuclease activity of CMoRE mutant 4A against T4 phage and *E. coli* genomic DNA. Source data are provided with this paper.

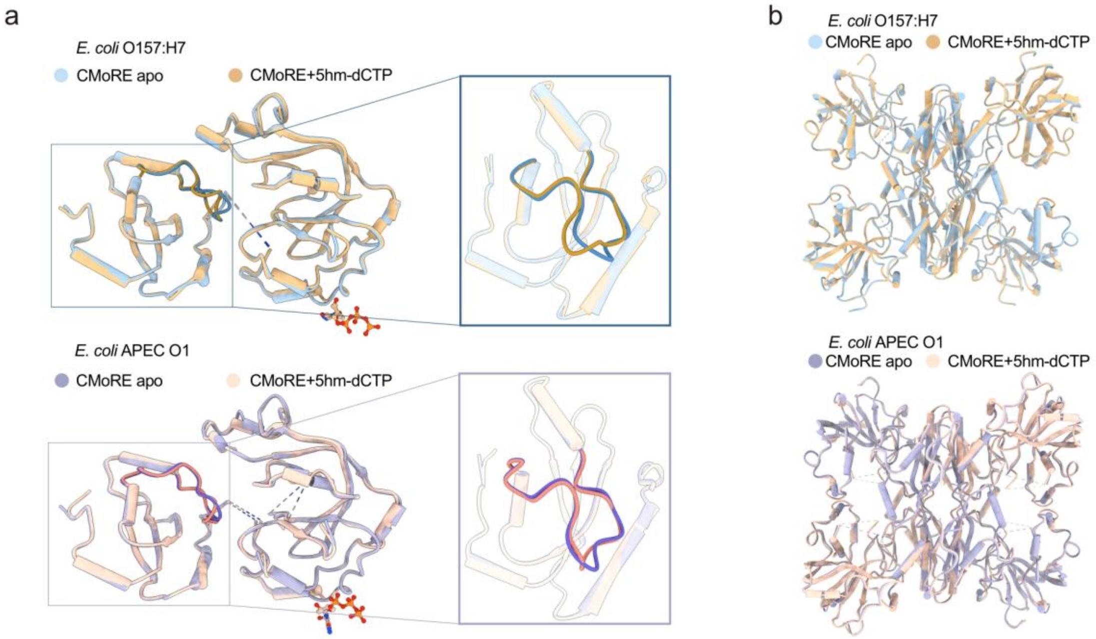

Supplementary Figure 7. Structural comparisons between apo and ligand-bound states of CMoRE. (a) Superposition of CMoRE protomers in apo and ligand-bound states. The autoinhibitory loops are highlighted by dark colors. (b) Superposition of CMoRE tetramers in apo and ligand-bound states.

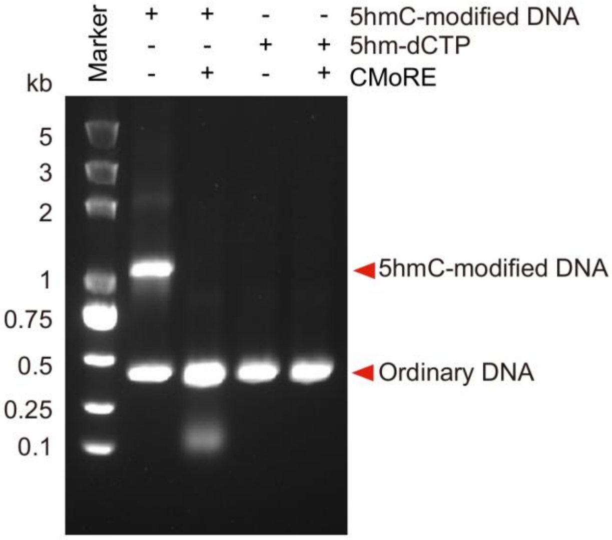

Supplementary Figure 8. The endonuclease activity of CMoRE against the ordinary DNA in the presence of 5hmC-modified DNA or 5hm-dCTP. Source data are provided with this paper.

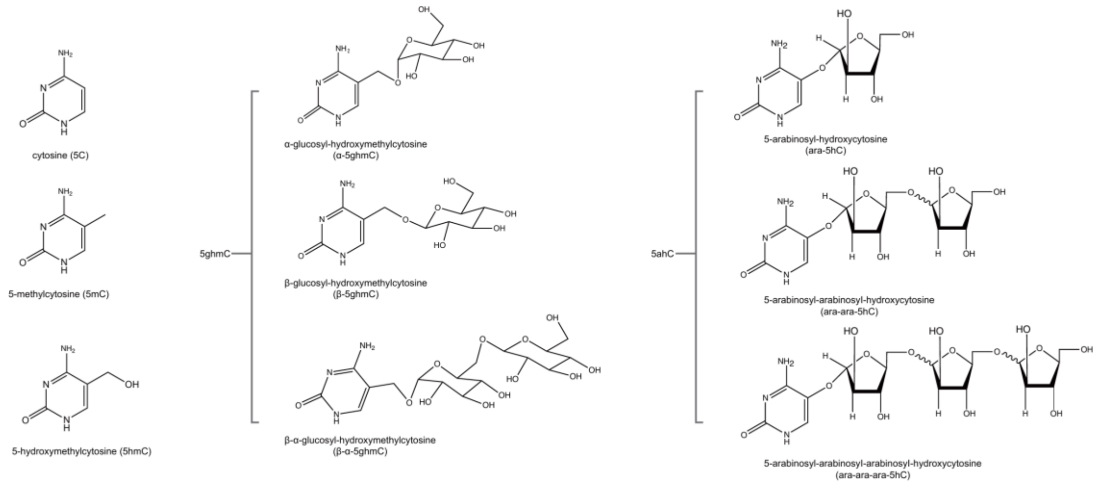

Supplementary Figure 9. Chemical structures of cytosine and its modifications.

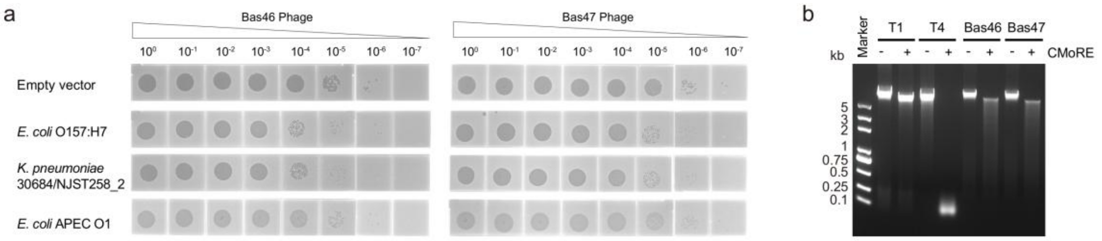

Supplementary Figure 10. Anti-phage activity of CMoRE defense systems from three bacterial species against phages Bas46 and Bas47. (a) Plaque formation assays. (b) Endonuclease activity of *E. coli* O157:H7 CMoRE against phages Bas46 and Bas47 genomic DNA. T1 and T4 phage genomic DNA are used as the negative and positive controls, respectively. Source data are provided with this paper.

***E. coli* O157:H7**

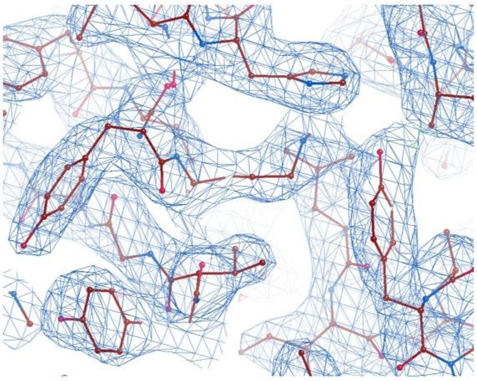

CMoRE

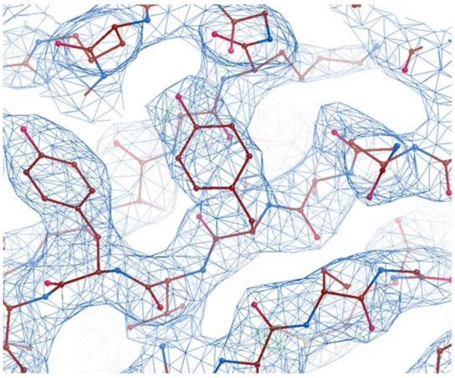

CMoRE+5hm-dCTP

***E. coli* APEC O1**

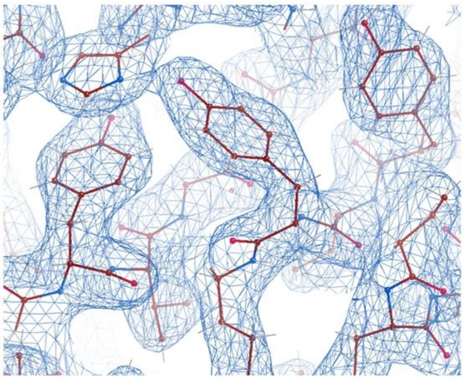

CMoRE

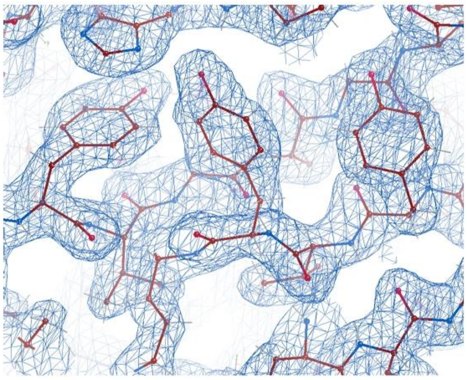

CMoRE+5hm-dCTP

Supplementary Figure 11. The electron density maps of each crystal structure in this study. The 2Fo-Fc maps (shown in blue) are contoured at 1 σ.

Supplementary Table 1. Data collection and refinement statistics.

	CMoRE (*E. coli* APEC O1)	CMoRE/5hm-dCTP (*E. coli* APEC O1)	CMoRE (*E. coli* O157:H7)	CMoRE/5hm-dCTP (*E. coli* O157:H7)
**PDB ID**	9U7U	9U7Z	9U75	9U8D
**Data collection**				
Space group	$P6_422$	$P6_422$	$P4_1$	$P4_1$
Cell dimensions				
a, b, c (Å)	123.3, 123.3, 82.9	124.7,124.7, 80.8	103.6,103.6, 125.8	103.6,103.6, 126.3
α, β, γ (°)	90.0, 90.0, 120.0	90.0, 90.0, 120.0	90.0, 90.0, 90.0	90.0, 90.0, 90.0
Resolution (Å)	38.7-2.2 (2.33-2.20)[a]	49.4-2.15 (2.19-2.15)	50-2.80 (2.87-2.80)	47.9-3.0 (3.05-3.0)
*R*meas (%)	10 (207)	11 (217)	13 (190)	17 (173)
$I/\sigma I$	33.7 (1.6)	37.7 (2.5)	21.2 (1.5)	15.7 (2.0)
CC (1/2)	1.0 (0.668)	0.975 (0.723)	0.991 (0.572)	0.995 (0.576)
Completeness (%)	99.9 (99.5)	99.1 (97.2)	100 (100)	100 (100)
Redundancy	15.7 (10.0)	38.1 (27.7)	13.8 (13.7)	13.9 (13.8)
**Refinement**				
Resolution (Å)	38.6-2.2	49.3-2.1	47.9-2.8	47.9-3.0
No. reflections	19,546	19,853	30,605	24,977
$R_{work/}$ R_{free} (%)	22.17/25.83	23.75/27.64	22.64/28.58	22.65/26.87
**B-factors**				
Protein	57	43	53	52
Ligand/ion	NA	59	NA	60
**Ramachandran**				
Favored (%)	97.92	97.5	97.54	95.79
Allowed (%)	2.08	2.5	2.46	4.21
Outlier (%)	0	0	0	0
**R.m.s deviations**				
Bond lengths (Å)	0.006	0.003	0.004	0.005
Bond angles (°)	0.834	0.629	0.664	0.778

[a]Highest resolution shell is shown in parenthesis.
